# Adjuvanted subunit intranasal vaccine reduces SARS-CoV-2 onward transmission in hamsters

**DOI:** 10.3389/fimmu.2025.1514845

**Published:** 2025-02-07

**Authors:** Yongjun Sui, Swagata Kar, Bhavna Chawla, Tanya Hoang, YuanKai Yu, Shannon M. Wallace, Hanne Andersen, Jay A. Berzofsky

**Affiliations:** ^1^ Vaccine Branch, National Cancer Institute, National Institutes of Health, Bethesda, MD, United States; ^2^ Bioqual Inc., Rockville, MD, United States; ^3^ Cancer Genetics Branch, National Cancer Institute, National Institutes of Health, Bethesda, MD, United States; ^4^ Experimental Pathology Laboratories, Inc., Sterling, VA, United States

**Keywords:** SARS-CoV-2 vaccine, mucosal vaccine, adjuvant subunit vaccine, onward transmission, mRNA vaccine

## Abstract

**Introduction:**

Most COVID-19 vaccine trials have focused on recipient protection, not protection of their contacts, a critical need. As a subunit intranasal COVID-19 vaccine reduced nasopharyngeal virus more than did an intramuscular (IM) vaccine, we hypothesized that this vaccine might reduce onward transmission to others.

**Methods:**

We vaccinated hamsters with either the IM-administrated licensed mRNA vaccine twice or one dose of mRNA IM followed by adjuvanted subunit intranasal vaccine. 24 hours after SARS-CoV-2 challenge, these animals were housed with naïve recipients in a contactless chamber that allows airborne transmission.

**Results:**

Onward airborne transmission was profoundly blocked: the donor and recipients of the intranasal vaccine-boosted group had lower oral and lung viral loads (VL), which correlated with mucosal ACE2 inhibition activity. Notably, in this head-to-head comparison of COVID-19 booster vaccines on SARS-CoV-2 onward transmission, we found that statistically significant viral reduction in the lung tissues and oral swabs was observed only in the intranasal S1 nanoparticle vaccine-boosted group, but not in the systemic mRNA vaccine-boosted group, suggesting the superior protection of this intranasal vaccine, which could act as an attractive vaccine booster candidate to complement the current licensed systemic vaccines.

**Discussion:**

Overall, our study strongly supports the use of the intranasal vaccine as a boost to protect not only the vaccinated person, but also people exposed to the vaccinated person, a key public health goal.

## Introduction

Blocking viral transmission is an important function of efficient vaccines. From a public health point of view, preventing SARS-CoV-2 transmission to other susceptible individuals is extremely critical. However, most COVID-19 vaccine clinical trials studied only safety and protection of the vaccine recipient, but not prevention of transmission to others. Indeed, the currently licensed SARS-CoV-2 vaccines are successful to alleviate COVID-19-related hospitalization and deaths, but less effective against acquisition of infection and onward transmission (1–3). Though studies on SARS-CoV-2 breakthrough infections suggested that vaccine breakthrough infections are less contagious than primary infections in unvaccinated individuals (4, 5), the effects of these vaccines on reducing transmissibility have not been well evaluated.

As SARS-CoV-2 transmission is mostly through the nasopharynx, mucosal immunity could potentially reduce or abort the SARS-CoV-2 replication at the portal of entry (nasopharynx) to prevent virus from being transmitted to others. Intranasal administration of current vaccines, however, led to inconsistent results against SARS-CoV-2 infections (6, 7). The adjuvant subunit mucosal vaccine, which induces vigorous mucosal immunity in the upper and lower respiratory tracts (8–10), and is more effective at clearing upper airway virus than a similar subunit vaccine given intramuscularly (IM), may have the potential to better reduce SARS-CoV-2 onward transmission. Here, we assessed whether the adjuvanted subunit vaccine (SARS-CoV-2 spike S1+S2 trimer of variants D614G and B.1.1.529 in DOTAP nanoparticles together with adjuvants Poly I:C, CpG and recombinant murine IL-15) delivered intranasally could protect from onward transmission of SARS-CoV-2 in a hamster model better than the systemic mRNA vaccine. As SARS-CoV-2 virus can be effectively transmitted among the hamsters, this represents a more natural dose and route of infection/transmission (11).

## Results and discussion

**Figure 1:**
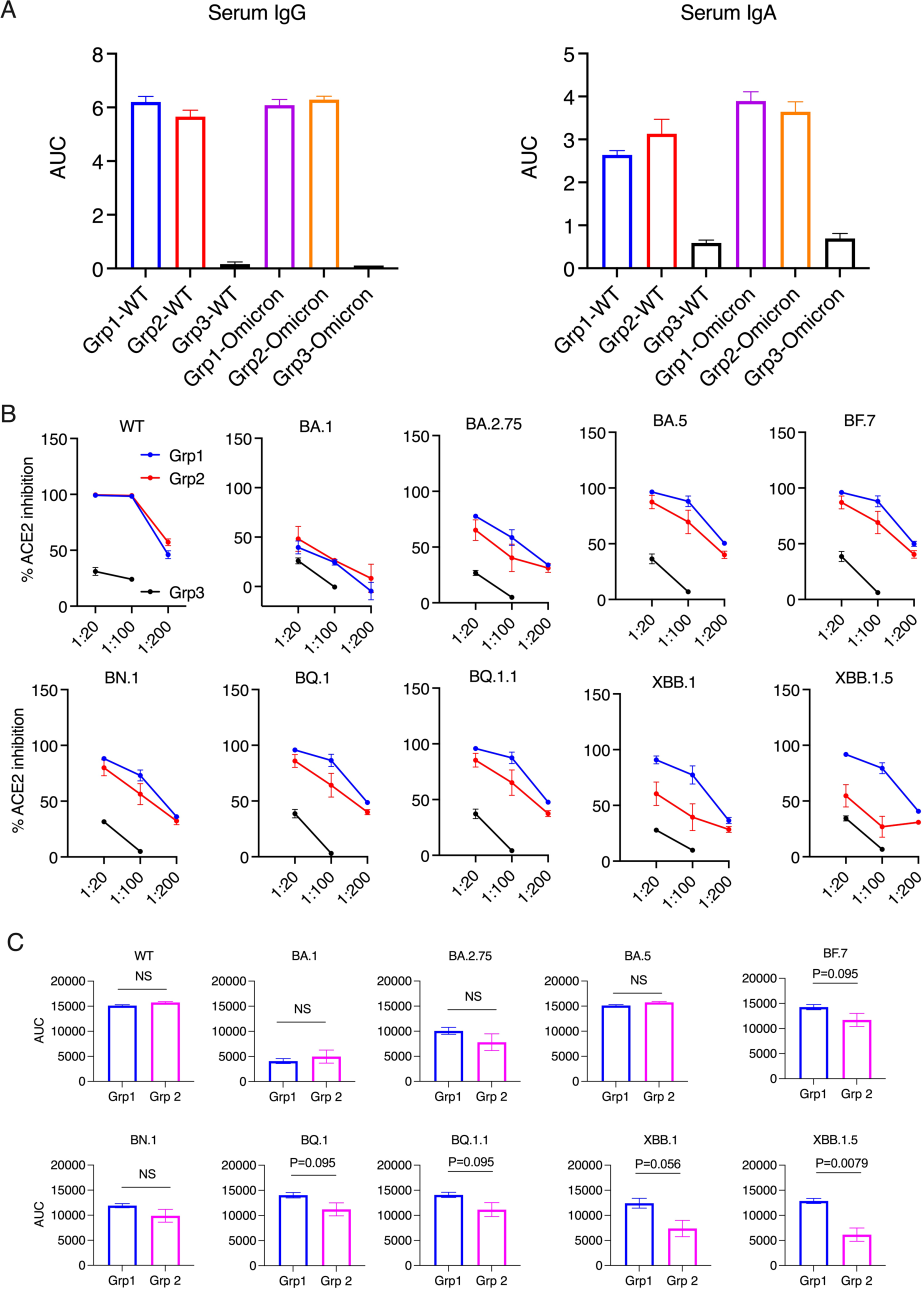
Serum antibody responses and ACE2 inhibition activity in the donor animals 2 weeks after the boost. **(A)**. Anti-spike IgG and IgA in serum samples were measured using ELISA. The serum samples were diluted from 1:100, 4-fold dilution, and 6 dilutions. Area under curve (AUC) of serum IgG and IgA are shown. **(B, C)**. The ACE2 inhibition activity against wild type (WT) and Omicron sub-strains in the serum samples of the donor animals (2 weeks after the boost). Area under curve (AUC) of serum ACE2 inhibition activities were calculated and compared between the two vaccinated groups **(C)**. Mann-Whitney tests were used for group comparisons.

To vaccinate the donor hamsters, we first primed IM two groups of male animals (n=5/group) with Moderna bivalent mRNA COVID-19 vaccine (Moderna Therapeutics, MA) to mimic the fact that many individuals have already been vaccinated with at least one or more doses of systemic vaccines. Male animals were chosen as they are more likely to have higher viral load (VL) and more severe disease (10) (**Figure 1A**). Three weeks later, Group 1 was boosted with the same mRNA vaccine (IM), while Group 2 was boosted intranasally (IN) with CP-15 adjuvanted (CpG+polyI:C+IL15) spike protein in DOTAP. Four weeks later, all groups, including a naïve control group (Group 3), were intranasally challenged with SARS-CoV-2 virus.

**Figure 2:**
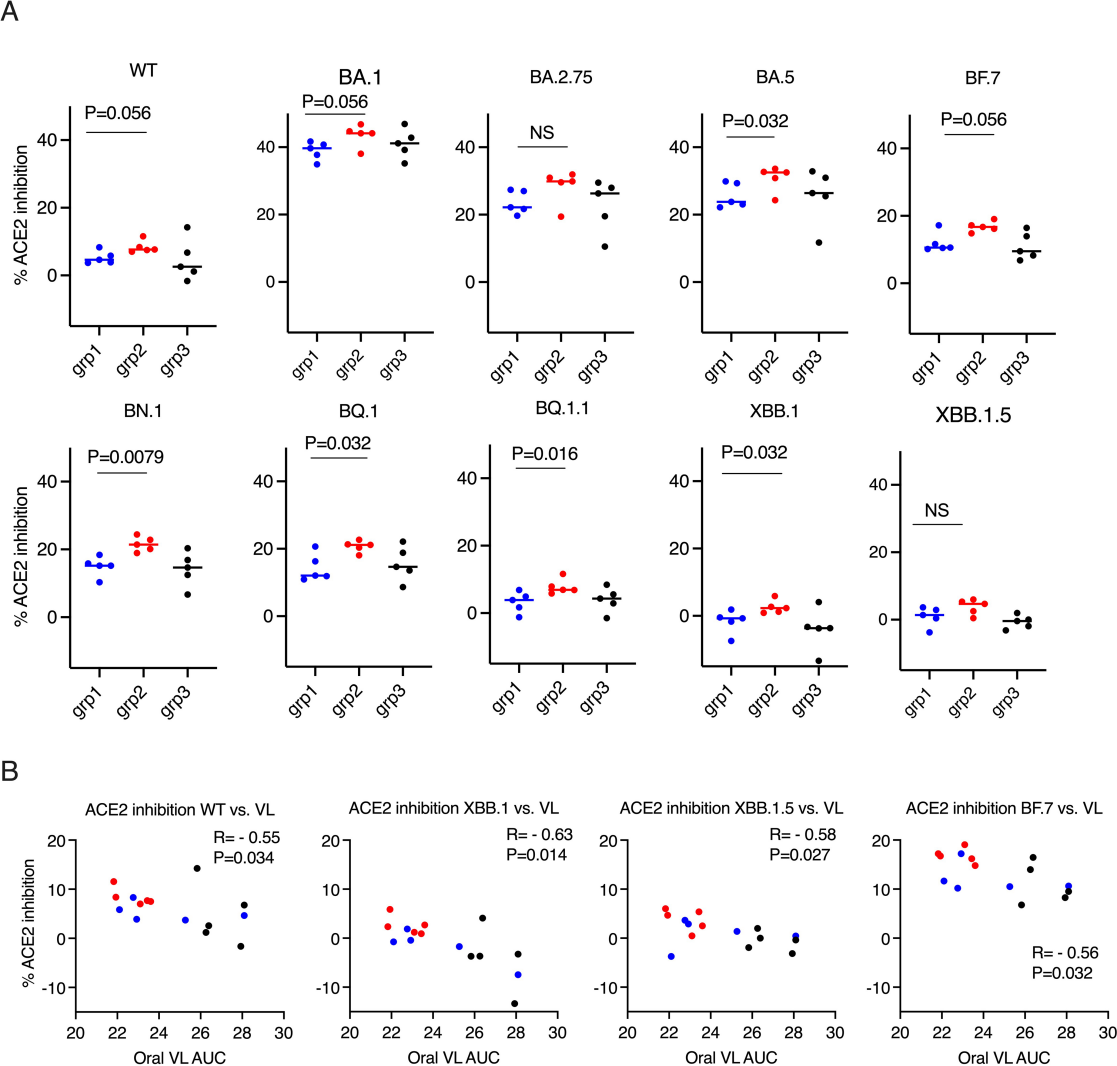
ACE2 inhibition activity was inversely correlated with viral load (VL) in the oral swabs. **(A)**. the ACE2 inhibition activity against wild type (WT) and Omicron sub-strains in the oral swabs of the donor animals (2 weeks after the boost). Mann-Whitney tests were used for group comparisons. **(B)**. Spearman’s correlations between ACE2 inhibition activity against WT/Omicron sub-strains XBB. 1/XBB. 1.5/BF.7 and the viral load (VL) in oral swabs. The groups are color coded, with Blue, red, and black denoting group 1-3 donor animals respectively. R and P values of Spearman’s correlations are shown.

Before studying forward transmission, we wanted to assess the immunity induced by the intranasal vs IM vaccine boosters in the immunized animals to be used as donors in the transmission study. In the serum, total IgG and IgA against WT SARS-CoV-2 (the original Wuhan or WA strain) and against Omicron were comparable, whereas the control animals had negligible binding IgG or IgA (**Figure 1A**) (ELISA titrations are shown in **Supplementary Figure S1**). In the previous studies using macaques and mice (refs 8, 9, and 15), we have extensively characterized the immunogenicity of the CP-15 adjuvanted mucosal vaccine, and found that it induced both systemic and mucosal antigen-specific humoral and cellular immune responses (**Supplementary Figure S2**). Here, we assessed the ACE2 inhibition activity (a surrogate neutralizing antibody assay (12), that is antibody blocking binding to the cell’s receptor for SARS-CoV-2, the Angiotensin converting enzyme 2) against the original Wild type (WT, WA or Wuhan) and 9 Omicron sub-strains in the serum and oral swabs of the vaccinated animals. In the serum, Group 1 had similar or higher levels of ACE2 inhibition activity compared to Group 2 (**Figures 1B, C**). Only the titers against XBB.1.5 were significantly higher in Group 1 vs Group 2 (p=0.0079), and the titers against BF.7(P=0.095), BQ.1 (p=0.095), BQ.1.1(P=0.095), XBB.1 (p=0.056) showed trends of higher titers in Group 1 than in Group 2 (**Figure 1C**). However, in contrast, in the oral swabs (**Figure 2A**), it is important to note that the opposite was true, i.e., the mucosal ACE2 inhibition titers were consistently higher for group 2 than for group 1 against most of the variants. This difference was significant for BA.5 (p = 0.032), BN.1 (p = 0.0079), BQ.1 (p = 0.032), BQ1.1 (p = 0.016), XBB.1(P=0.032), and a strong trend for WT (p = 0.056), BA.1 (p = 0.056), and BF.7 (p=0.056). These multiple corroborative results support that conclusion that the mucosal vaccine was more effective at inducing neutralizing activity in the mucosal secretions than in the serum. These results corroborate for these donor animals what we have seen with live virus neutralization assays in our previous studies in hamsters and rhesus macaques (8–10, 13). Likewise, these earlier studies confirm that neutralizing activity is detectable by live virus neutralization assays in both non-human primates and hamsters, supporting the findings by ACE2 binding inhibition here (See **Supplementary Figure S2**). Moreover, ACE2 inhibition activity in oral swabs was inversely correlated with oral VLs two weeks after viral oropharyngeal challenge, suggesting the mucosal immunity might play a more important role in reducing viral infections (**Figure 2B**). We also observed that some of the animals in naïve control group had higher ACE2 binding inhibition activities. We speculate that a high variability of background activity for fluids collected from mucosal tissues such as oral cavity might account for this. Nevertheless, these data might also explain the observation that asymptomatic or mildly symptomatic breakthrough infections occurred in subjects who maintained their systemic antibody and T-cell responses (14). We also note that the findings in **Figures 1**, **2** that the mucosal boost increases only mucosal antibody, not serum antibody, above the levels induced by the IM boost provides indirect evidence suggesting that the mucosal nanoparticles do not leak out to the systemic immune system.

**Figure 3:**
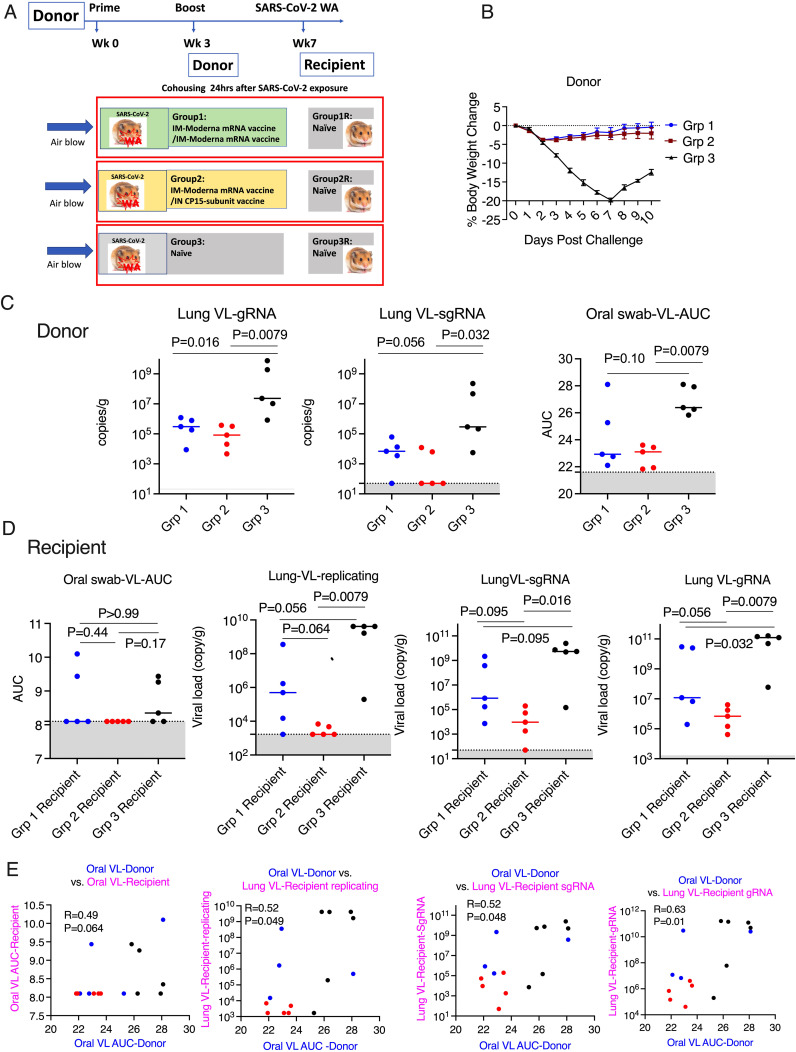
Mucosal vaccine prevented SARS-CoV-2 onward transmission in hamsters. **(A)**. Schematic of SARS-CoV-2 transmission study. **(B)**. Body weight change in the donor hamsters after the challenge of SARS-CoV-2 Washington strain. **(C)**. Area under curve (AUC) of viral load (VL) in oral swabs at Days 1, 2, 5, and 7, and sgRNA/gRNA VL in the lung at Day 10 after viral challenge in the donor hamsters. **(D)**. AUC of VL in oral swabs at Day 1, 2, and 3, replicating, sgRNA/gRNA VL in the lung at Day 3 after housing in the recipient hamsters. **(E)**. the Spearman’s correlations between the VL in the donor animals and the VL in the recipient animals. Mann-Whitney and Spearman analyses were used for group comparisons and correlations. The groups are color coded, with Blue, red, and black denoting group 1-3 donor and recipient animals respectively. Dotted lines and grey shading indicate the lower limit of detection.

After this characterization of vaccine-induced immune response and protection of the intended “donor” animals, we could set up the main study, to examine forward transmission from infected vaccinated animals to naïve recipients exposed only by the airborne route. Three groups of naïve hamsters (n=5/group) were used as recipients to assess the transmission rate 24 hours after the SARS-CoV-2 viral challenge. In each contact-free cage, under BSL3 containment, one donor animal was housed with one naive recipient animal for 8 hours with unidirectional air flow, through a permeable membrane that prevented physical touching or transfer of secretions, but allowed airborne transmission of virus, from the donor to the recipient. The donor animals were monitored for weight loss, oral VL for an additional 8 days after housing, while the recipient groups were necropsied at day 3 post viral exposure to examine the VL in the lung tissues (**Figure 3A**). Neither donor vaccine group showed significant weight loss, suggesting both vaccines as a booster could provide sufficient protection against disease (**Figure 3B**). Despite the small number of animals, both vaccine donor groups demonstrated significantly reduced gRNA (genomic RNA interpreted as input virus) in the lung tissues (at day 10) compared to the naïve group (P=0.016 and P=0.0079 for Group 1 and 2 respectively). However, statistically significant reduction of sgRNA (subgenomic RNA, which is present only in replicating virus and taken as a measure of replicating virus) was observed in Group 2 (P=0.032) with the mucosal boost, but not in Group 1 (P=0.056). Moreover, both the median gRNA, which were 3.04X10^5/g in Group 1, 8.36X10^4/g in Group 2, and 2.32X10^7/g in the naïve group, and the median sgRNA, which were 6900/g in Group 1, 50/g in Group 2, and 2.93X10^5 in the naïve group, follow a similar trend. In the oral swabs, we observed again that Group 2 (P=0.0079), but not Group 1 (P=0.10), showed significant viral reduction (**Figure 3C**) (AUC in **Figure 3** is based on time course data in **Supplementary Figure S3** upper row).

For recipient groups after airborne exposure, none of the 5 animals housing with Group 2 had detectable oral VLs, indicating complete protection, while 3 out of 5 animals housing with Group 3 and 2 out of 5 housing with Group 1 showed oral VLs (**Figure 3D**) (Oral virus AUC based on time course data presented in **Supplementary Figure S3**, lower row). In the lung, the replicating virus titer (measured by tissue-culture infectious dose-50 or TCID50, therefore virus particles capable of infecting cells in culture) and sgRNA were significantly reduced only in the animals housed with Group 2 (mucosal boost) compared to those cohoused with control Group 3 (P=0.0079 for replicating virus titer, and P=0.016 for sgRNA), while no significant protection was observed in the animals housed with Group 1 (P=0.056 and P=0.095, respectively; **Figure 3D**). The median replicating virus titer was 4.97X10^5 in recipients housed with IM Group 1, but only 1.68X10^3 in recipients housed with mucosal Group 2 (2.5 logs lower), compared to 4.12X10^9 in recipients housed with the naïve group, and the sgRNA was 8.48X10^5 in recipients housed with group 1, but only 9.44X10^3 (2 logs lower) in those housed with mucosal Group 2, compared to 5.37X10^9 in naïve control. Nevertheless, both systemic and mucosal vaccines demonstrated significant reduction of SARS-CoV-2 gRNA in recipients (P=0.032 for group 1, and P=0.0079 for Group 2), with median gRNA in Group 1 and 2 recipients of 1.19X10^7, but only 6.87 X10^5 respectively, compared to 1.23X10^11 in naïve group. Thus, even though the conventional IM route vaccination does not prevent viral transmission, the viral loads in the recipients were lower, compared to these not vaccinated but not nearly as low as in recipients housed with mucosally boosted animals. Though a statistical trend due to the small number of animals (P=0.056 for gRNA and replicating virus titers, and P=0.095 for sgRNA), the hamsters in the recipient group housing with group 2 consistently had a log or two lower median gRNA, sgRNA, and replicating viral load levels in the lungs and oral swabs than those housed with group 1 (**Figure 3D**). Indeed, only the mucosal vaccine group 2 recipients (that received the intranasal S1 protein+ adjuvant nanoparticles) had a consistently significantly lower transmission than the control group in all 3 measures of lung VL, Lung replicating VL (p = 0.0079), Lung sgRNA (p= 0.016) and lung gRNA (p = 0.0079), whereas group 1 IM vaccine was significant only in the last of these (p = 0.056, 0.095 and 0.032, respectively). Also, in the oral swabs, only the recipients of group 2 were all completely negative (**Figure 3D** left). By these criteria, the mucosal vaccine (with the intranasal S1 protein+adjuvant nanoparticles) was more consistently effective and quantitatively more effective against air-borne transmission to naïve hamsters than the IM vaccine. These finding supports the greater efficacy of the intranasal vaccine for preventing onward transmission to naïve hamsters. Histopathological exams revealed that both vaccines were effective in reducing SARS-CoV-2-related microscopic findings in the lung when compared to Group 3 animals; Group 2 was most effective with the lowest incidence of findings (**Table 1** and **Supplementary Figure S4**). Note that Groups 1-3 (donor animals) were necropsied on day 10 after viral challenge, whereas the recipients Groups 4-6 were necropsied on day 3 after co-housing to detect transmission, before much pathological change in the lungs developed. Additionally, both vaccines were effective in eliminating the transmission of SARS-CoV-2 related microscopic findings in the lung of untreated co-housed animals (**Table 1** and **Supplementary Figure S4**), although the day 3 necropsy of the recipients was too early to see much inflammation. We also found that oral VL in the donors was weakly correlated with lung and oral VLs in the recipients (P=0.064 for oral VL, P=0.049 and 0.048 and 0.01 for lung replicating VL and sgRNA and gRNA; **Figure 3E**), which was consistent with the previous finding that onward viral transmission is multifactorial, and the level of infectious virus in the donor oropharynx was one of the key parameters (15). Overall, the data indicated that as a booster, the mucosal intranasal vaccine with the S1 protein+adjuvant nanoparticles provided substantially better blockage against onward transmission than the systemic mRNA vaccine did.

**Table 1:** SARS-CoV-2-related microscopic findings in the lung of the donor (necropsied at Day 10 post challenge) and the recipient animals (necropsied at Day 3 post housing) *.

Groups	1	2	3	Groups	1 Recipient	2 Recipient	3 Recipient
Animals/Group	5	5	5^a^	Animals/Group	5	5	5
**Inflammation, mixed or mononuclear cell, alveolar or bronchoalveolar**	3	–	5	**Inflammation, mononuclear cell, alveolar**	–	–	1
minimal	3	–	–	minimal	–	–	1
mild	–	–	4				
moderate	–	–	–				
marked	–	–	1				
**Inflammation, mononuclear cell, vascular/perivascular**	–	–	5	**Inflammation, mononuclear cell, vascular/perivascular**	–	–	3
minimal	–	–	3	minimal	–	–	2
mild	–	–	2	mild	–	–	1
**Fibrosis or fibroplasia, pleural**	–	–	2				
minimal	–	–	1				
mild	–	–	1				
**Syncytial cell**	–	–	5				
minimal	–	–	5				
**Hyperplasia, bronchiolo-alveolar or alveolar**	5	1	5				
minimal	3	–	–				
mild	2	1	–				
moderate	–	–	3				
marked	–	–	2				
**Hyperplasia, endothelial**	–	–	2				
minimal	–	–	2				
Hemorrhage	–	–	2				
minimal	–	–	–				
mild	–	–	2				
**Hypertrophy, mesothelial cell**	–	–	1				
minimal	–	–	–				
mild	–	–	1				

*Findings were graded 1-5, depending upon severity. Microscopic findings, if applicable, are correlated with macroscopic observations. For severity grades, equivalent numbered grades are 1 = minimal, 2 = mild, 3 = moderate, 4 = marked, 5 = severe.

One limitation of this study is that we could not evaluate IgG subtypes or the cell-mediated immunity (CMI) (T cell response), which is important for the durability of the vaccine, as the reagents for measuring IgG subtypes and T cell responses in hamsters are limited or largely non-existing. However, in our recent study, we found that the same CP-15 adjuvanted mucosal vaccine as a booster induced robust CMI responses in mouse models (16). As most current vaccines prevent COVID-19 disease but do not prevent initial infection and spread to others, it is important to focus on developing vaccines that block SARS-CoV-2 onward transmission. Previous studies using adenovirus type 5 SARS-CoV-2 mucosal vaccines showed reducing viral transmission compared to naïve donors (17). However, the data to compare the protection capacity against onward transmission with currently licensed systemic vaccines are lacking. Here we did a head-to-head comparison of a subunit mucosal vaccine versus a licensed mRNA vaccine boost for their ability to induce mucosal immunity and subsequently prevent airborne SARS-CoV-2 onward transmission from vaccinated donors to naïve recipients. Our results demonstrated that CP-15 adjuvanted subunit mucosal intranasal vaccine, as a booster, mediated at least as good, and by most parameters, better protection against onward airborne transmission, compared to the licensed systemic mRNA SARS-CoV-2 vaccine (more consistent and quantitatively stronger protection compared to the control group than the systemic vaccine). The induction of mucosal naso-oropharyngeal immunity was a correlate of protection (**Figure 2B**) and showed consistently higher titers of ACE2-inhibiting antibody in the mucosal secretions after mucosal boost than the systemic vaccine against multiple viral variants of concern. Thus, this mucosal vaccine, along with other mucosal vaccines, could act as attractive vaccine booster candidates to complement the current licensed systemic vaccines to limit SARS-CoV-2 onward transmission and fulfill a critical public health need that has not been addressed.

## Materials and methods

### Animals

All animal studies were approved by the BIOQUAL Animal Care and Use Committee (Rockville, MD). Thirty male Syrian golden hamsters (Envigo), 8–10 weeks old, were housed and conducted in compliance with all relevant regulations.

### Vaccination

The hamsters were grouped randomly into 6 groups (N=5/group). Group 1-3 were the donor groups, and Group 4-6 were the recipient groups. 10 µg/dose Moderna COVID-19 bivalent vaccine (containing mRNA for both original and Omicron BA.4/BA.5 variants) (in 100µl) was given to Group 1 -2 intramuscularly at Day 0. On Day21, Group1 got the same dose/route of Moderna COVID-19 vaccine. Group 2 received intranasally CP-15 adjuvanted mucosal vaccine, which was composed of 20 µg of SARS-CoV-2 Spike S1+S2 trimer protein (10 µg D614G + 10 µg B.1.1.529) (40589-V08H8, 40589-V08H26, Sino Biological. Inc.), mixed with 20 µg of D-type CpG oligodeoxynucleotide (vac-1826-1, *In vivo*Gen), 40 µg of Poly I:C (vac-pic, *In vivo*Gen), and 20 µg of recombinant murine IL-15 (210-15, PeproTech) in 20 µl of DOTAP (11 811 177 001, Roche Inc.)((1,2-dioleoyl-3-trimethylammonium propane) is a cationic lipid used for DNA transfection of cells. We use it as an adjuvant by mixing the antigen and TLR ligand adjuvants and cytokines with DOTAP to form micellular nanoparticles that protect the components from degradation and deliver them to cells.). The variants used were the most recent available for animal use at the time of the study. Moreover, the purpose of the study was to test proof of principle that the mucosal vaccine would be more effective at reducing the risk of onward transmission, so the specific strain of SARS-CoV-2 was not critical to this goal. For the intranasal procedures, the hamsters were sedated with Ketamine (80µg/kg)/Xylazine(5µg/kg), and 50 uL/nare, total 100uL vaccine was administrated per hamster. The dosing material was more likely to penetrate further into the respiratory tract, getting to the lungs by using this procedure (sedation and size of the inoculum).

### Viral challenge and viral transmission

Four weeks after the last vaccination, Group 1-3 (Group 3 was the naïve control group) were challenged with 6x10^3^ PFU SARS-CoV-2 WAS-CALU-3 (LOT: 12152020-1235, BEI Resources). The animals were sedated, and virus challenge was given intranasally with 50 µl/nare as described before (10). Body weights were monitored before and after the viral challenge. 24hrs after the viral challenge, each animal from Group1-3 was housed with one naïve hamster from Group 4-6 respectively. The housing was in a single contract-free transmission chamber as described before (17). The chamber was designed so that the airflow was unidirectional: from the infected donor animal to the naïve recipient one. Animals were housed 1:1 with a transmission divider for 8 hours, then single housed. The donor animals were monitored for an additional 8 days for body weights, oral VLs. At Day10, the donor animals were necropsied, and lung viral loads were measured. For recipient groups, body weights and oral VLs was monitored and at Day3, the animals were necropsied, and lung VLs were measured.

### ELISA and ACE2 inhibition assay

ELISA and ACE2 inhibition assay were performed as described (10, 12). The V-PLEX-SARS-CoV-2 Panel 32 (ACE2) Kit was used according to the manufacturer’s instructions using a Sector Imager 2400 (Meso Scale discovery). Serum samples (diluted 20-, 100-, and 200-fold), or oral samples (2-fold dilution) were added into the pre-coated V-plex plates. ACE2 binding and detection reagents were sequentially added.

### Viral load measurements

TCID50 assays were used to measure live virus as described before (10). Briefly, 20 µL of sample was 10-fold serially diluted and added to Vero TMPRSS2 cells, cultured in DMEM + 2% FBS + Gentamicin at 37°C, 5.0% CO2 for 4 days. Virus stock of known infectious titer was included in the assay as a positive control, while medium only served as a negative control. Cytopathic effect (CPE) was inspected, and the TCID50 value was calculated using the Read-Muench formula. SARS-CoV-2 RNA levels were assessed by reverse transcription PCR at BIOQUAL, Inc. as previously described (8). RNA was extracted from oral swab and homogenized lung tissue samples. Subgenomic/viral RNA using different primer/probe sets, targeting the viral E gene mRNA or the viral nucleocapsid respectively, was measured. VLs are shown as copies per swab for oral samples, and copies per gram for lung tissues, with a cutoff value of 50 copies.

### Histopathological exams

Groups 1-3 were euthanized on Day 10 post viral challenge. Recipient groups were euthanized on Day 3 post co-housing. At necropsy, the left lung was collected and placed in 10% neutral buffered formalin for histopathologic analysis. Tissue sections were trimmed and processed to hematoxylin and eosin (H&E) stained slides, all slides were examined by a board-certified pathologist and recorded using the Pristima 7.5.0 Build 8 version computer system.

### Statistical analysis

Statistical analyses were performed using Prism version 9. Oral swab viral load was presented as area under curve (AUC) values. Mann-Whitney and Spearman analyses were used for group comparisons and correlations. All statistical tests were 2-tailed.

## Data Availability

The original contributions presented in the study are included in the article/**Supplementary Material**. Further inquiries can be directed to the corresponding author.

## References

[1] Jung J Kim JY Park H Park S Lim JS Lim SY. Transmission and infectious SARS-coV-2 shedding kinetics in vaccinated and unvaccinated individuals. JAMA Netw Open. (2022) 5:e2213606. doi: 10.1001/jamanetworkopen.2022.13606 35608859 PMC9131744

[2] Riemersma KK Haddock LA 3rd Wilson NA Minor N Eickhoff J Grogan BE. Shedding of infectious SARS-CoV-2 despite vaccination. PloS Pathog. (2022) 18:e1010876. doi: 10.1371/journal.ppat.1010876 36178969 PMC9555632

[3] Salvatore PP Lee CC Sleweon S McCormick DW Nicolae L Knipe K. Transmission potential of vaccinated and unvaccinated persons infected with the SARS-CoV-2 Delta variant in a federal prison, July-August 2021. Vaccine. (2023) 41:1808–18. doi: 10.1016/j.vaccine.2022.11.045 PMC9744684 36572604

[4] Abu-Raddad LJ Chemaitelly H Ayoub HH Tang P Coyle P Hasan MR. Relative infectiousness of SARS-CoV-2 vaccine breakthrough infections, reinfections, and primary infections. Nat Commun. (2022) 13:532. doi: 10.1038/s41467-022-28199-7 35087035 PMC8795418

[5] Mostaghimi D Valdez CN Larson HT Kalinich CC Iwasaki A. Prevention of host-to-host transmission by SARS-CoV-2 vaccines. Lancet Infect Dis. (2022) 22:e52–8. doi: 10.1016/S1473-3099(21)00472-2 PMC8439617 34534512

[6] Cokaric Brdovcak M Materljan J Sustic M Ravlic S Ruzic T Lisnic B. ChAdOx1-S adenoviral vector vaccine applied intranasally elicits superior mucosal immunity compared to the intramuscular route of vaccination. Eur J Immunol. (2022) 52:936–45. doi: 10.1002/eji.202249823 PMC9087383 35304741

[7] Madhavan M Ritchie AJ Aboagye J Jenkin D Provstgaad-Morys S Tarbet I. Tolerability and immunogenicity of an intranasally-administered adenovirus-vectored COVID-19 vaccine: An open-label partially-randomised ascending dose phase I trial. EBioMedicine. (2022) 85:104298. doi: 10.1016/j.ebiom.2022.104298 36229342 PMC9550199

[8] Sui Y Li J Zhang R Prabhu SK Elyard HA Venzon D. Protection against SARS-CoV-2 infection by a mucosal vaccine in rhesus macaques. JCI Insight. (2021) 6:1–15. doi: 10.1172/jci.insight.148494 PMC8262352 33908897

[9] Sui Y Li J Andersen H Zhang R Prabhu SK Hoang T. An intranasally administrated SARS-CoV-2 beta variant subunit booster vaccine prevents beta variant replication in rhesus macaques. PNAS Nexus. (2022) 1:pgac091. doi: 10.1093/pnasnexus/pgac091 35873792 PMC9295201

[10] Sui Y Andersen H Li J Hoang T Bekele Y Kar S. Protection from COVID-19 disease in hamsters vaccinated with subunit SARS-CoV-2 S1 mucosal vaccines adjuvanted with different adjuvants. Front Immunol. (2023) 14:1154496. doi: 10.3389/fimmu.2023.1154496 37020550 PMC10067881

[11] Port JR Yinda CK Riopelle JC Weishampel ZA Saturday TA Avanzato VA. Infection- or vaccine mediated immunity reduces SARS-CoV-2 transmission, but increases competitiveness of Omicron in hamsters. Nat Commun. (2022) 14:6592. doi: 10.1101/2022.07.29.502072 PMC10584863 37852960

[12] Tan CW Chia WN Qin X Liu P Chen MI Tiu C. A SARS-CoV-2 surrogate virus neutralization test based on antibody-mediated blockage of ACE2-spike protein-protein interaction. Nat Biotechnol. (2020) 38:1073–8. doi: 10.1038/s41587-020-0631-z 32704169

[13] Sui Y Andersen H Li J Hoang T Minai M Nagata BM. SARS-CoV-2 mucosal vaccine protects against clinical disease with sex bias in efficacy. Vaccine. (2024) 42:339–51. doi: 10.1016/j.vaccine.2023.11.059 PMC10843685 38071106

[14] Rovida F Cassaniti I Paolucci S Percivalle E Sarasini A Piralla A. SARS-CoV-2 vaccine breakthrough infections with the alpha variant are asymptomatic or mildly symptomatic among health care workers. Nat Commun. (2021) 12:6032. doi: 10.1038/s41467-021-26154-6 34654808 PMC8521593

[15] Puhach O Meyer B Eckerle I. SARS-CoV-2 viral load and shedding kinetics. Nat Rev Microbiol. (2023) 21:147–61. doi: 10.1038/s41579-022-00822-w PMC9716513 36460930

[16] Li J Hsu KS Howe SE Hoang T Xia Z Berzofsky JA. Sex-biased immunogenicity of a mucosal subunit vaccine against SARS-CoV-2 in mice. Front Immunol. (2024) 15:1386243. doi: 10.3389/fimmu.2024.1386243 38835757 PMC11148259

[17] Langel SN Johnson S Martinez CI Tedjakusuma SN Peinovich N Dora EG. Adenovirus type 5 SARS-CoV-2 vaccines delivered orally or intranasally reduced disease severity and transmission in a hamster model. Sci Transl Med. (2022) 14:eabn6868. doi: 10.1126/scitranslmed.abn6868 35511920 PMC9097881

