# Supplementary material for: Adjuvanted subunit intranasal vaccine reduces SARS-CoV-2 onward transmission in hamsters
Source: Front Immunol. 2025 Feb 7;16:1514845. doi: 10.3389/fimmu.2025.1514845 (PMC11841495; doi:10.3389/fimmu.2025.1514845)
Supplement: Supplementary file 1 [file DataSheet1.pdf]

Supplementary figure 1. Spike-specific IgG and IgA responses against wild type (WT) and Omicron sub-strains in the serum samples of the vaccinated and naïve animals (2 weeks after the boost).

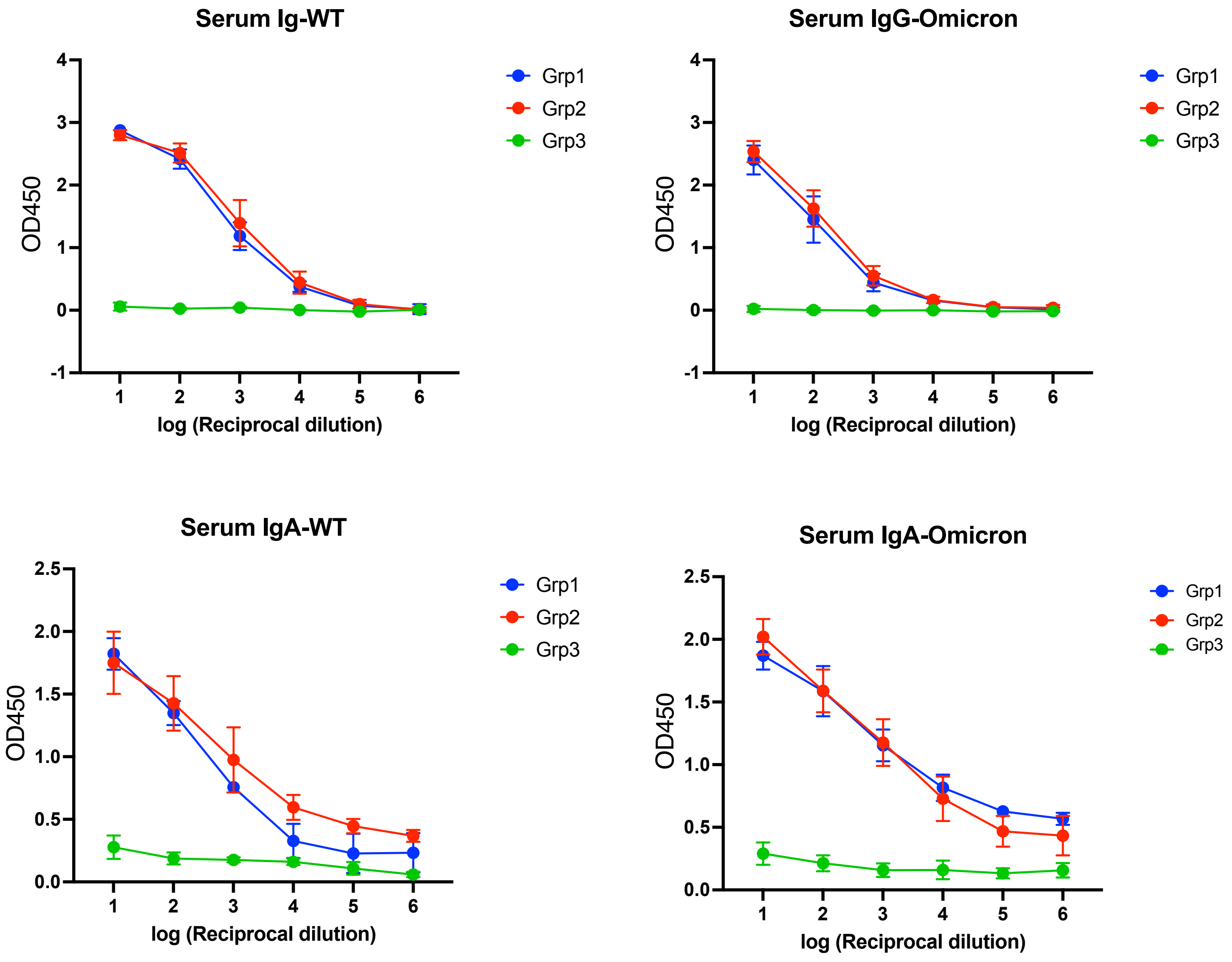

Supplementary figure 2. Summary immunogenicity data of CP15-adjuvanted SARS-CoV-2 mucosal vaccine from published papers. a-b from macaques *appropriate credit: “Protection against SARS-CoV-2 infection by a mucosal vaccine in rhesus macaques” published in JCI Insight. 2021 Apr 28;6(10) by Sui et al (https://pubmed.ncbi.nlm.nih.gov/33908897/),used under CCBY 4.0: (https://creativecommons.org/licenses/by/4.0/). No warranty or liability of the original authors or publisher are implied by the re-publication of this material. No warranty or liability of the original authors or publisher are implied by the re-publication of this material. while c-d from mice: https://pubmed.ncbi.nlm.nih.gov/38835757/(ref 8 and 15).

a.

Grp1: Alum-adjuvanted systemic vaccine

Grp2: CP15-adjuvanted mucosal vaccine

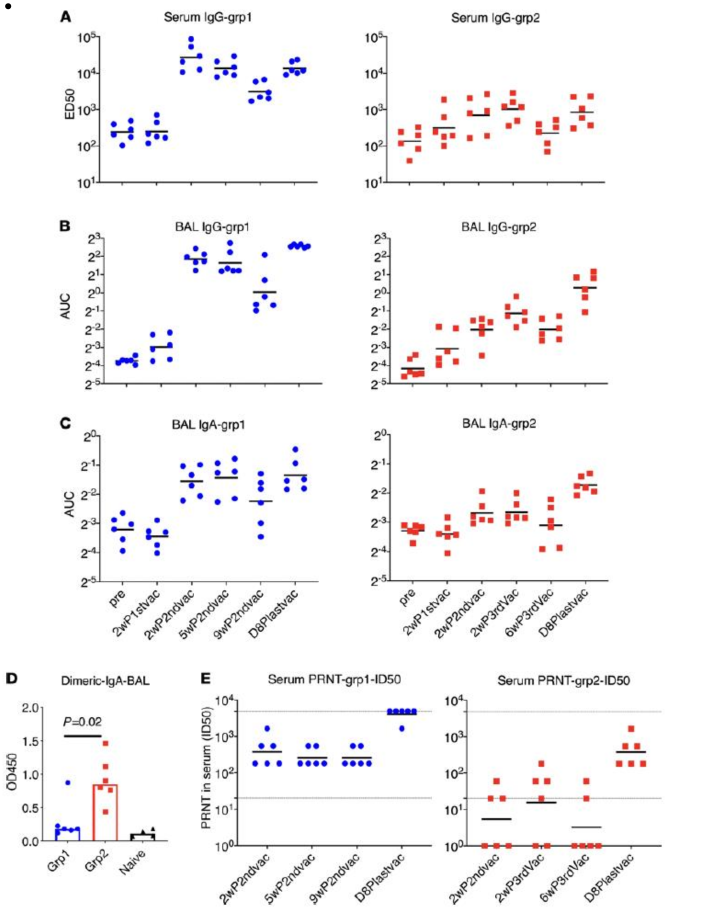

Figure 2. Spike-specific humoral immune responses in PBMC and bronchoalveolar lavage samples of the vaccinated animals. The ED_{50} of S1-specific IgG in serum (**A**) and the AUC of S1-specific IgG and IgA in bronchoalveolar lavage (BAL) (**B** and **C**) were measured during the whole course of vaccination. Dimeric IgA responses in BAL at day 8 after last vaccination (**D**) and PRNT (neutralizing) titers against live virus (**E**) in the serum samples were measured. BAL samples fror naive animals (n = 4) were included (**D**) to serve as a negative control to show the baseline. The Mann-Whitney U test was used to assess the difference between groups 1 and 2 (**D**). Short lines show geometric means. Dashed lines show the lower and upper assay limits. n = 6 for group 1 and 2. 2wP2ndvac, 2 weeks after second vaccine dose; 5wP2ndvac, 5 weeks after second vaccine dose; 9wP2ndvac, 9 weeks after second vaccine dose; 2wP3rdvac, 2 weeks aft third vaccine dose; 6wP3rdvac, 6 weeks after third vaccine dose; D8Plastvac, day 8 after last vaccination.

b.

Grp1: Alum-adjuvanted systemic vaccine

Grp2: CP15-adjuvanted mucosal vaccine

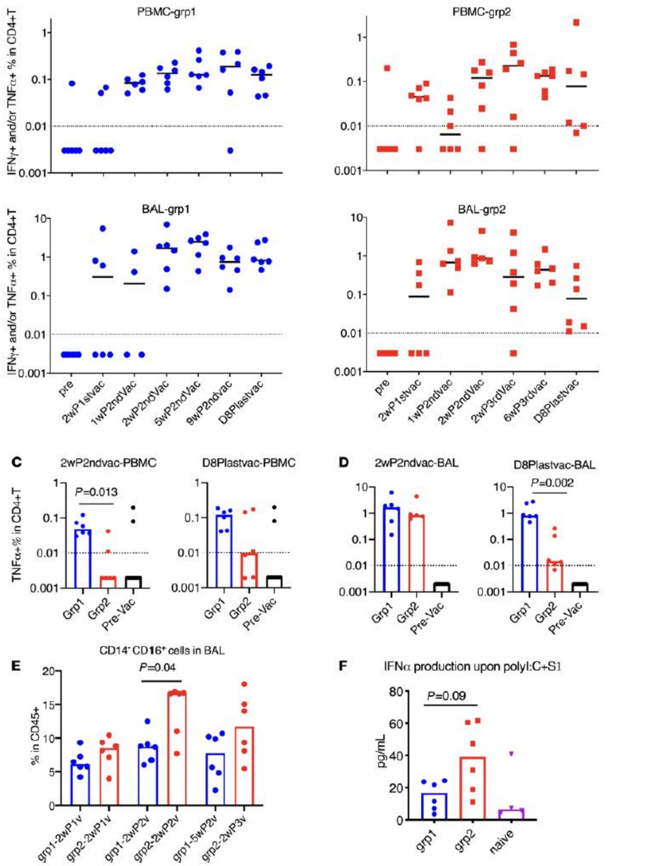

Figure 3. Spike-specific CD4+ T cell responses and trained immunity in PBMC and BAL samples of the vaccinated animals. Intracellular cytokine staining assays in responses to spike protein S1 were measured during the whole course of vaccination in PBMC (**A**) and BAL (**B**) samples. Spike-specific TNF-α+CD4+ T cell responses of different groups in PBMC (**C**) and BAL (**D**) samples at week 2 after second vaccination and day 8 after last vaccination were compared. PBMC and BAL samples from prevaccinated naive animals (n = 12) were included (**C** and **D**) to serve as negative control to show the baseline. The kinetics of CD14-CD16+ (monocyte or possibly NK) subsets were measured in the BAL samples of the vaccinated animals after 18 hours of PMA+ ionomycin stimulation (**E**). IFN-α was measured in the supernatant of BAL samples after 18 hours of Poly I:C plus S1 stimulation (**F**). Medians are shown. Serum from naive animals (n = 4) was included (**F**) to serve as negative control to show the baseline. Mann-Whitney U tests were used to compare the differences between groups (**D–F**). Dashed lines are the threshold for positive responses. n = 6 for groups 1 and 2.

c.

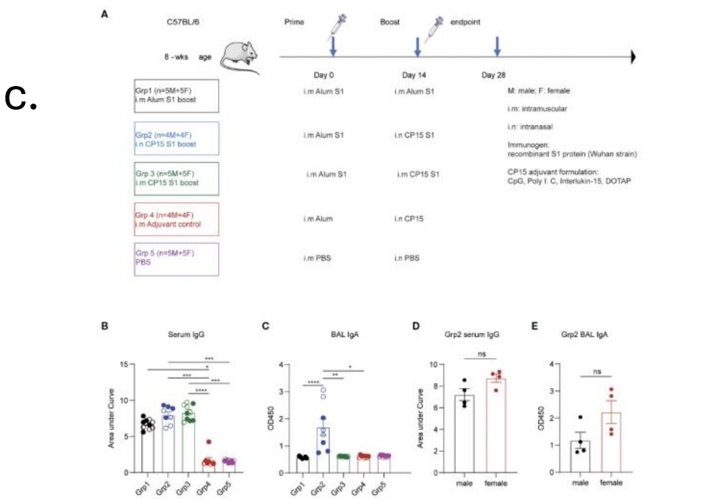

FIGURE 1

Vaccines induced spike-specific humoral responses in sera and bronchoalveolar lavage. **(A)** experimental timeline, treatment groups, animal numbers, and vaccination protocol. **(B)** IgG Ab titters in sera collected at two weeks post boost vaccination (day 28). **(C)** BAL IgA Ab responses at day 28 day. **(D)** comparison of male and female IgG responses. **(E)** BAL IgA Ab in males and females. Empty circle dots: female; Solid circle dots: male in **(B, C)**. Non-parametric one-way ANOVA using the Kruskal-Wallis test, followed by corrected Dunn's multiple comparisons tests, was performed to analyze serum IgG and BAL IgA responses in group1–5 **(B, C)**. Data are representative of two independent experiments (n=4 or 5 per sex). The nonparametric Mann-Whitney test was used to compare sex differences in grp2 Ab responses **(D, E)**. The significance levels for p-values were denoted as follows: 0.1234 (ns), 0.0332 (*), 0.0021(**), 0.0002 (***), <0.0001 (****).

d.

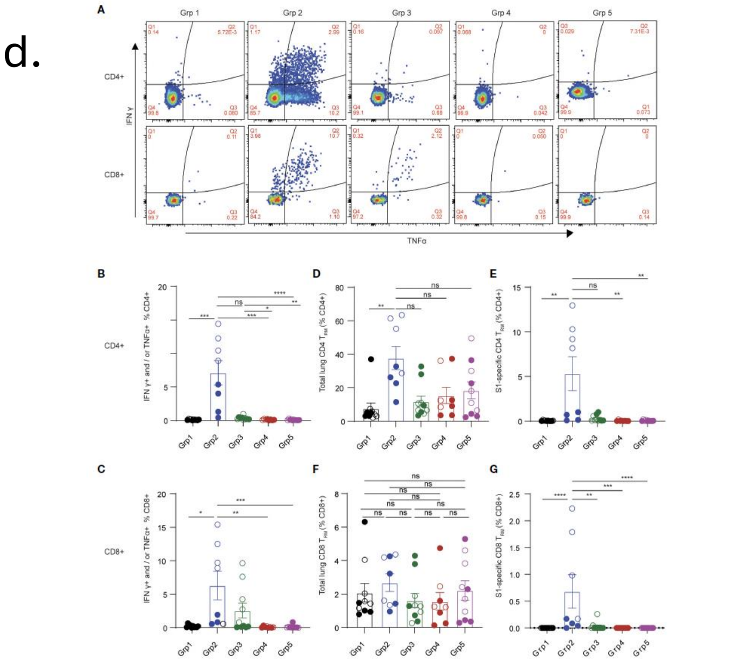

FIGURE 2

Spike-specific T cell responses in the lungs of C57BL/6 mice. **(A)** representative flow cytometry plots showing spike-specific T cell responses for each animal group. **(B)** spike-specific CD4+ T cell responses. **(C)** spike-specific lung CD8+ T_{RM}. **(D, E)** total, and spike-specific lung CD4+ T_{RM}, respectively. **(F, G)** total, and spike-specific lung CD8+ T_{RM}, respectively. Empty circle dots: female; Solid circle dots: male in **(B–G)**. Data are representative of two independent experiments (n=4 or 5 per sex). Non-parametric one-way ANOVA using the Kruskal-Wallis test followed by corrected Dunn's multiple comparisons tests was conducted in Prism to analyze cellular responses across groups 1–5. The significance levels for p-values were denoted as follows: 0.1234 (ns), 0.0332 (*), 0.0021(**), 0.0002 (***), <0.0001 (****) **(B–G)**.

Supplementary figure 3. Oral Viral loads after SARS-CoV-2 challenge.

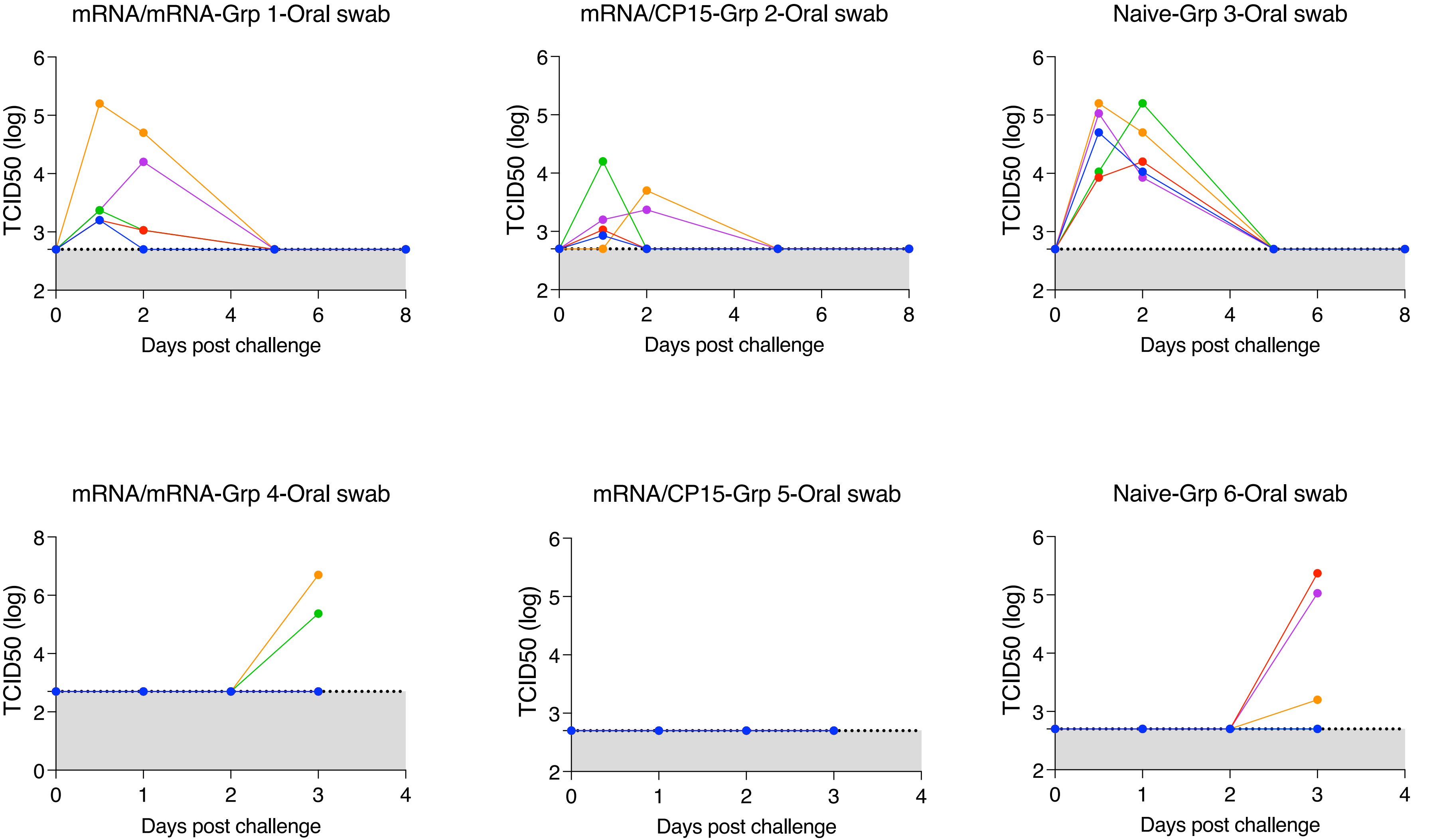

Supplemental figure 4. Histopathology of representative animals from group1-6. For the upper 3 panels (donor animals), both mRNA vaccine + mRNA vaccine (Group 1, left image) and mRNA vaccine +CP15-adjuvanted S1 subunit vaccine (Group 2, middle image) treatments were effective in reducing SARSCoV-2 (WA strain) related microscopic findings in the lung when compared to untreated / challenged animals (Group 3, right image); mRNA vaccine + CP15-adjuvanted S1 subunit vaccine (Group 2) was most effective with the lowest incidence of findings. SARS-CoV-2 (WA strain) microscopic findings are characterized by darkly basophilic consolidated multifocal areas (arrows) to coalescing areas. In untreated / challenged animals (Group 3, right image), the majority of the lung section represented is affected with microscopic findings. For the lower 3 panels (recipient animals), both mRNA vaccine + mRNA vaccine (Group 4) and mRNA vaccine + CP15-adjuvanted S1 subunit vaccine (Group 5) treatments were effective in eliminating the transmission of SARSCoV-2 related microscopic findings in the lung of untreated co-housed animals, Groups 4 (top image) and 5 (middle image), respectively, when compared to animals co-housed (Group 6, bottom image) with SARSCoV-2 (WA strain) challenged animals (Group 3). SARS-CoV-2 (WA strain) microscopic findings are characterized by darkly basophilic consolidated multifocal areas (arrow).

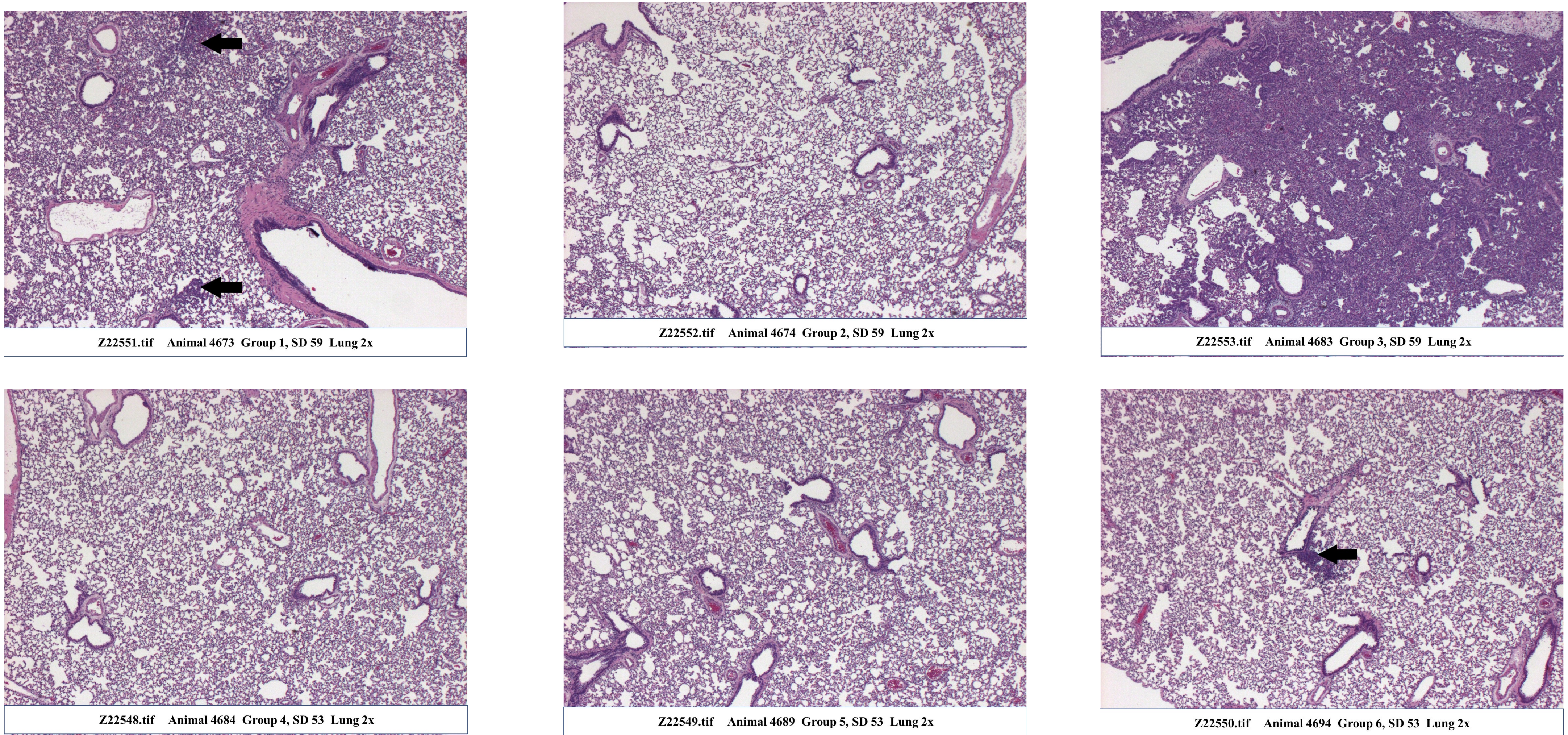

Z22551.tif Animal 4673 Group 1, SD 59 Lung 2x

Z22552.tif Animal 4674 Group 2, SD 59 Lung 2x

Z22553.tif Animal 4683 Group 3, SD 59 Lung 2x

Z22548.tif Animal 4684 Group 4, SD 53 Lung 2x

Z22549.tif Animal 4689 Group 5, SD 53 Lung 2x

Z22550.tif Animal 4694 Group 6, SD 53 Lung 2x
